# Pedal medial arterial calcification in diabetic foot ulcers: A significant risk factor of amputation and mortality

**DOI:** 10.1111/1753-0407.13527

**Published:** 2024-04-07

**Authors:** Lihong Chen, Dawei Chen, Hongping Gong, Chun Wang, Yun Gao, Yan Li, Weiwei Tang, Panpan Zha, Xingwu Ran

**Affiliations:** ^1^ Department of Endocrinology & Metabolism West China Hospital, Sichuan University Chengdu China; ^2^ Innovation Center for Wound Repair, Diabetic Foot Care Center West China Hospital, Sichuan University Chengdu China; ^3^ International Medical Center Ward, Department of General Practice West China Hospital, Sichuan University Chengdu China; ^4^ Department of Clinical Research Management West China Hospital, Sichuan University Chengdu China

**Keywords:** amputation, diabetic foot ulcer, mortality, pedal medial arterial calcification

## Abstract

**Aims:**

Pedal medial arterial calcification (MAC) is frequently observed in individuals with diabetic foot ulcers (DFUs). However, the impact of pedal MAC on individuals with DFUs remains uncertain. The main aim of this study was to evaluate the association between pedal MAC with amputation and mortality outcomes.

**Methods:**

A prospective, observational cohort study was conducted at West China Hospital from January 2012 to December 2021. Logistic regression analyses, Kaplan–Meier survival method, and Cox proportional hazards models were employed to evaluate the relationship between pedal MAC and amputation as well as mortality.

**Results:**

A total of 979 patients were enrolled in the study. Peripheral artery disease (PAD) was observed in 53% of patients with DFUs, and pedal MAC was found in 8%. Over a median follow‐up of 46 (23–72) months, foot amputation was performed on 190 patients, and mortality occurred in 246 patients. Pedal MAC showed a significant association with amputation both in unadjusted analysis (odds ratio [OR] = 2.98, 95% confidence interval [CI] = 1.86–4.76, *p* < .001) and after adjusting sex, age, albumin levels, hemoglobin levels, and diabetic retinopathy status (OR 2.29, 95% CI 1.33–3.93, *p* = .003). The risk of amputation was found to be twofold higher in individuals with PAD and pedal MAC compared to those with PAD alone (OR 2.05, 95% CI 1.10–3.82, *p =* .024). Furthermore, the presence of pedal MAC was significantly associated with an increased risk of mortality (*p* = .005), particularly among individuals with DFUs but without PAD (HR 4.26, 95% CI 1.90–9.52, *p* < .001), rather than in individuals presenting with both DFUs and PAD.

**Conclusion:**

The presence of pedal MAC is significantly associated with both amputation and mortality in individuals with DFUs. Moreover, pedal MAC could provide additional value to predict amputation other than PAD.

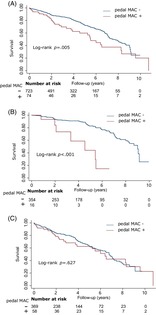

## Graphical abstract

Highlights

- The impact of pedal medial arterial calcification (MAC) on individuals with diabetic foot ulcers (DFUs) remains uncertain.
- This study revealed a significant association between pedal MAC and both amputation and mortality in individuals with DFUs. Furthermore, pedal MAC demonstrated additional predictive value for amputation beyond that of PAD alone.
- The findings underscore the importance of preventing and reversing MAC in individuals with DFUs.

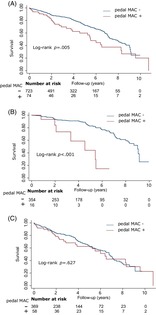

## 1 INTRODUCTION

Diabetic foot ulcers (DFUs) impose a significant burden on both individuals and health care service systems worldwide. Approximately one quarter of individuals with diabetes would develop a foot ulcer at some point in their lifetime.^1^ It is estimated that around 20% of these ulcers will ultimately result in the need for amputation.^2^ Amputations not only seriously affect mobility and quality of life but also represent one of the most dreaded complications associated with diabetes. Moreover, they have been shown to reduce life expectancy. Approximately one quarter of individuals succumb within 30 days following amputation, nearly half expire within 1 year, and up to 80% perish within 5 years.^3^ Therefore, preventing amputation remains one of the most pressing objectives in managing DFUs.

However, the precise pathological mechanisms underlying gangrene and ulcers remain elusive.^4^, ^5^ Peripheral neuropathy and peripheral artery disease (PAD) are widely acknowledged as prominent risk factors for DFUs. It is well established that PAD exhibits a robust association with major cardiovascular events, amputations, and mortality. However, the association between PAD, as measured by ankle brachial index, and limb complications is not as robust as that with cardiovascular events.^6^, ^7^ Hence, it is evident that there are other significant contributors to limb complications apart from atherosclerotic PAD.

The concept of medial arterial calcification (MAC) was initially introduced in 1903 by Mönckeberg, shortly after the invention of radiographic imaging. MAC represents a distinct vascular disorder separate from atherosclerosis, primarily affecting the tunica media of small and midsized arteries.^8^ In recent years, several histological studies have unveiled a close association between MAC and limb complications related to PAD.^5^ In the study conducted by O'Neill et al, it was observed that 72% of individuals with critical limb ischemia exhibited involvement of MAC in their lower limb arteries.^9^ Another study by Narula and colleagues revealed a frequent occurrence of MAC‐related thrombus in vascular occlusion below the knee.^10^ Hence, it is plausible to speculate that MAC might play a role in the pathogenesis of adverse limb events associated with PAD.

The presence of MAC is predominantly associated with diabetes, chronic kidney disease, and aging,^11^ thereby increasing the susceptibility of individuals with DFUs to this condition, particularly affecting the microvasculature in the lower extremities. Although there is inconsistency in defining calcification criteria, nearly 50% of diabetic patients with foot disease exhibit evidence of MAC.^12^, ^13^ In our unpublished study, we also observed a high prevalence of pedal MAC in individuals with DFUs. Moreover, MAC is increasingly acknowledged as a significant risk factor for progressive hemodynamic impairment due to small artery disease.^11^

In the studies conducted by Vartanian and colleagues, it was observed that the MAC score of the foot exhibited a significant association with both major amputation^14^ and hemodynamic alterations following revascularization in individuals with chronic limb‐threatening ischemia.^15^ In a recent study, So and colleagues conducted an assessment of the DFU characteristics in individuals with stages 3b–5 chronic kidney disease (CKD) and also investigated the associated risk of major amputation.^16^ They discovered that the presence of severe MAC significantly increased the likelihood of major amputation (odds ratio [OR], 4.46; 95% confidence interval [CI], 1.43–13.88; *p* = .010) within the CKD population.

However, the existence of this association in the population with DFUs remains unexplored. Furthermore, it is imperative to investigate whether the presence of pedal MAC could exert additional impact on limb events in individuals with DFUs. Therefore, the objective of this study was to evaluate the correlation between pedal MAC and limb amputation. Additionally, we aimed to investigate whether pedal MAC, in conjunction with PAD, could exacerbate the prognosis of individuals with DFUs. Furthermore, we sought to assess the association of pedal MAC with all‐cause mortality.

## 2 METHODS

### 2.1 Subjects

The present study was a prospective, observational cohort study conducted at West China Hospital from January 2012 to June 2023. The study was approved by the Ethics Committee on Biomedical Research at West China Hospital of Sichuan University, and informed consent were obtained from each individual participant. This study adhered to the Strengthening the Reporting of Observational Studies in Epidemiology (STROBE) guideline. A total of 979 individuals with DFUs were enrolled at our diabetic foot care center. Radiographic examination was performed on the affected foot or both feet for all patients.

### 2.2 Interventions

All patients were provided with standard care for DFUs, which included off‐loading, anti‐infection measures, debridement, local wound management, negative‐pressure wound therapy, and autologous platelet‐rich gel application based on the clinical judgment of the attending clinician. Given the frequent coexistence of various chronic disease, such as hypertension, coronary artery disease, and CKD among individuals with DFUs, it is common for these individuals to undergo metabolic control and receive treatment for their comorbidities. Demographics information, clinical characteristics, and laboratory data were extracted from medical records. Follow‐up on amputation and mortality was conducted via telephone between 2022 and 2023.

### 2.3 The definition of peripheral artery disease and amputation

PAD was defined as an ankle brachial index (ABI) score below 0.9 or more than 50% stenosis in duplex ultrasound, and amputation encompassed both major and minor amputation.

### 2.4 The assessment of pedal medial arterial calcification

All patients underwent anteroposterior foot X‐ray imaging at baseline, and some also received either lateral or oblique views. The dorsalis pedis, lateral plantar artery, metatarsal, hallux, and nonhallux digital arteries were evaluated for the presence of MAC. Arterial calcification was determined by the presence of tram‐track sign. Additionally, we measured the lengths of these arteries to assess the extent of MAC. A score of 1 will be assigned if the length of the dorsalis pedis, lateral plantar artery and metatarsal artery exceeds 2 cm, or if the length of the hallux and nonhallux digital artery exceeds 1 cm. The total score for pedal MAC was calculated by summing up the scores from all five arteries.^14^, ^17^ A patient with a score higher than 1 point was classified as having pedal MAC.

### 2.5 Statistical analysis

We presented the data as either the number of individuals (%), or as mean ± SD, or median (interquartile range [IQR]). When conducting this analysis, any missing data were disregarded. Considering the significant impact of PAD on limb amputation, we introduced a novel variable called PAD_MAC to represent both the presence of PAD and pedal MAC. All individuals with DFUs were classified into four groups: normal, pedal MAC alone, PAD only, and pedal MAC + PAD group. Appropriate statistical tests including analysis of variance test, Kruskal–Wallis test, and chi‐square test were conducted to compare between‐group differences. Because the accurate timing of amputation events could not be obtained, univariate and multivariate logistic regression analyses were used to evaluate the association between pedal MAC and amputation risk. We further conducted an analysis in the subset of individuals with DFUs and PAD to evaluate the additional impact of pedal MAC on amputation risk. The Kaplan–Meier method was employed to construct survival curves for pedal MAC and mortality, followed by a log‐rank test. Unadjusted and adjusted Cox models were used to examine the association between pedal MAC and mortality. Additionally, we performed subgroup analyses based on the presence or absence of PAD. Statistical analysis was carried out using Stata version 13, considering *p* values <.05 as statistically significant.

## 3 RESULTS

The baseline characteristics of the study population, categorized based on the presence of pedal MAC and PAD, were presented in Table 1. A total of 979 individuals with DFUs were included in this analysis. The average age was 65.2 ± 12.1 years, with women accounting for 35% of the cohort. The majority patients had type 2 diabetes (971 patients, 99.2%), and only eight individuals had type 1 diabetes. The mean duration of diabetes was 11.49 ± 7.74 years. PAD was observed in 53% patients with DFUs, and pedal MAC was present in nearly 8% of patients. Coexistence of both pedal MAC and PAD was found in 6% of patients with DFUs, whereas the occurrence of isolated pedal MAC without PAD was rare (2%). A total of 65 (6%) patients had end‐stage renal disease (ESRD), and 197 patients (20%) had coronary artery disease.

**TABLE 1:** Baseline characteristics of patients with DFUs.

	Total (*n* = 979)	Normal (*n* = 436)	Pedal MAC alone (*n* = 20)	PAD (*n* = 458)	Pedal MAC + PAD (*N* = 65)	*p* value
Sex (male)	636 (65%)	285 (65%)	14 (70%)	280 (61%)	57 (88%)	<.001
Age (years)	65.2 ± 12.1	59.9 ± 12.2	58.6 ± 13.2	70.6 ± 9.5	64.9 ± 10.2	<.001
BMI (kg/m^2^)	23.3 ± 3.4	23.7 ± 3.7	25.1 ± 5.7	22.9 ± 3.0	23.2 ± 2.7	.002
Smoking (%)	497 (51%)	219 (51%)	7 (35%)	228 (50%)	43 (66%)	.044
ESRD (%)	65 (6%)	16 (4%)	6 (30%)	14 (3%)	29 (45%)	<.001
Hyperparathyroidism (%)	20 (2%)	1 (0.2%)	2 (10%)	7 (2%)	10 (15%)	<.001
Duration of diabetes (years)	11.49 ± 7.74	10.41 ± 7.29	13.45 ± 8.75	12.07 ± 7.99	13.98 ± 7.58	<.001
Coronary artery disease (%)	197 (20%)	42 (10%)	2 (10%)	127 (28%)	26 (40%)	<.001
Hypertension (%)	664 (68%)	244 (56%)	12 (60%)	354 (77%)	54 (83%)	<.001
Parathyroid hormone (mmol/L)	4.62 (2.37–6.38)	4.22 (2.98–5.75)	5.36 (4.62–19.32)	4.79 (3.57–6.49)	6.00 (3.69–22.72)	<.001
25OH‐vitamin D (nmol/L)	37.81 ± 17.76	38.82 ± 18.19	28.27 ± 14.34	37.35 ± 17.14	37.99 ± 19.48	.102
Calcium (mmol/L)	2.22 ± 0.29	2.22 ± 0.22	2.13 ± 0.41	2.23 ± 0.36	2.21 ± 0.18	.592
Phosphate (mmol/L)	1.18 ± 0.29	1.19 ± 0.30	1.29 ± 0.29	1.14 ± 0.24	1.31 ± 0.38	<.001
Alkaline phosphatase (mmol/L)	80 (65–106)	80.0 (63–106)	80 (65–128)	80 (65–104)	87 (70.5–113)	.251
Total cholesterol (mmol/L)	3.78 ± 1.14	3.75 ± 1.16	3.36 ± 0.78	3.84 ± 1.14	3.68 ± 0.98	.210
HDL cholesterol (mmol/L)	1.07 ± 0.37	1.11 ± 0.42	0.99 ± 0.19	1.03 ± 0.32	0.99 ± 0.35	.003
LDL cholesterol (mmol/L)	2.02 ± 0.87	2.01 ± 0.90	1.85 ± 0.72	2.06 ± 0.86	1.96 ± 0.82	.601
Triglycerides (mmol/L)	1.54 ± 0.98	1.53 ± 0.92	1.40 ± 0.54	1.56 ± 1.08	1.51 ± 0.67	.877
HbA1c (%)	8.35 ± 2.21	8.59 ± 2.56	8.66 ± 2.95	8.20 ± 1.82	7.75 ± 1.83	.007

Abbreviations: BMI, body mass index; CAD, coronary artery disease; ESRD, end‐stage renal disease; HbA1c, hemoglobin A1c; HDL, high‐density lipoprotein; LDL, low‐density lipoprotein; MAC, medial arterial calcification; PAD, peripheral artery disease.

The study revealed significant disparities in gender, age, body mass index, smoking status, ESRD presence, hyperparathyroidism occurrence, duration of diabetes mellitus, coronary artery disease, hypertension, parathyroid hormone levels, phosphate concentrations, high‐density lipoprotein cholesterol levels, and hemoglobin A1c values. The proportion of individuals with ESRD was significantly higher in the pedal MAC + PAD group than that in PAD only group (45% vs 3%, *p* < .001). Additionally, the mean age in the pedal MAC + PAD group was younger than that in the PAD‐only group (64.9 ± 10.2 vs 70.6 ± 9.5, *p* < 0.001) as shown in Table 1.

### 3.1 Amputation

Among the 979 individuals with DFUs, a total of 190 patients underwent foot amputation during a median follow‐up period of 46 months (IQR 23–72 months). The amputation rate was found to be 13.99% in the normal group, whereas it was higher at 20.00%, 20.96%, and significantly elevated at 44.62% in the pedal MAC‐alone, PAD‐only, and pedal MAC + PAD groups, respectively (*p* < 0.001). There was no significant difference of amputation between pedal MAC‐only and PAD‐only group (*p* = 0.918). However, patients with both pedal MAC and PAD exhibited a significantly higher amputation rate compared to those with only PAD (*p* < .001).

The risk factors for amputation were assessed using univariate logistic regression analysis, which revealed significant associations between amputation and gender, smoking status, ABI score, Wagner grade, coronary artery disease, peripheral artery disease, diabetic retinopathy, cholesterol levels, albumin levels, hemoglobin levels, pedal MAC presence, and PAD_MAC presence in patients with DFUs (Table 2). As demonstrated in Table 2, the presence of pedal MAC was found to be significantly associated with amputation in patients with DFUs (OR = 2.98, 95% CI = 1.86–4.76, *p* < .001). Even after adjusting for confounding factors such as sex, age, albumin, hemoglobin, and diabetic retinopathy, the association between pedal MAC and amputation remained significant (OR 2.29, 95% CI 1.33–3.93, *p* = .003).

**TABLE 2:** Univariate analysis of amputation in patients with diabetic foot ulcers.

	OR	95% CI	*p* value
Sex (male)	1.60	1.13–2.27	.009
Age	1.01	0.99–1.02	.279
Smoking	1.38	1.00–1.90	.049
ABI score	0.47	0.23–0.98	.044
Wagner grade	2.04	1.67–2.51	<.001
Coronary artery disease	1.61	1.12–2.33	.011
PAD	1.89	1.36–2.63	<.001
Diabetic retinopathy	1.36	1.07–1.73	.02
Cholesterol	0.84	0.72–0.97	.018
HDL cholesterol	0.69	0.44–1.07	.099
LDL cholesterol	0.83	0.69–1.01	.057
Albumin	0.97	0.95–0.99	.027
Hemoglobin	0.99	0.98–0.99	.001
Pedal MAC	2.98	1.86–4.76	<.001
PAD_MAC	1.45	1.24–1.69	<.001

Abbreviations: ABI, ankle brachial index; CI, confidence interval; HDL, high‐density lipoprotein; LDL, low‐density lipoprotein; MAC, medial arterial calcification; OR, odds ratio; PAD, peripheral artery disease; PAD_MAC, a variable generated as a representative of the presence of PAD and pedal MAC.

PAD is widely acknowledged as a significant risk factor for limb amputation. In this study, we also found a positive association between PAD and an increased risk of amputation (OR = 1.89, 95% CI = 1.36–2.63, *p* < .001). To investigate the potential impact of pedal MAC on limb outcomes in patients with PAD, we assessed the relationship between pedal MAC and amputation specifically within the subset of patients with DFUs. In the subset of patients with PAD, pedal MAC remained significantly associated with an increased the risk of amputation in both crude model (OR 3.03, 95% CI 1.77–5.20, *p* < .001) and after adjusting for sex, age, albumin, hemoglobin, and diabetic retinopathy (OR 2.14, 95% CI 1.14–4.02, *p* = .018). (Table 3).

**TABLE 3:** The risk factors of amputation in the PAD subset of patients with diabetic foot ulcers.

	Unadjusted		Adjusted^a^	
	OR (95% CI)	*p* value	OR (95% CI)	*p* value
Sex (male)	1.49 (0.97–2.31)	.071	1.19 (0.73–1.92)	.490
Age	0.98 (0.96–1.00)	.080	0.99 (0.97–1.02)	.708
Albumin	0.99 (0.96–1.02)	.431	0.99 (0.95–1.02)	.514
Hemoglobin	0.99 (0.99–1.00)	.267	0.99 (0.99–1.01)	.862
Diabetic retinopathy	1.49 (1.09–2.04)	.014	1.48 (1.05–2.09)	0026
Pedal MAC	3.03 (1.77–5.20)	<.001	2.14 (1.14–4.02)	.018

Abbreviations: CI, confidence interval; MAC, medial arterial calcification; OR, odds ratio; PAD, peripheral artery disease.

^a^ Adjusted for age, sex, albumin, hemoglobin, and diabetic retinopathy.

Incorporating the variable PAD_MAC, which serves as a representative measure for both PAD and pedal MAC, multivariate logistic regression analysis revealed a significant association between PAD_MAC and the occurrence of amputation (*p* value for trend: OR 1.42, 95% CI 1.19–1.70, *p* < .001). Patients with PAD and pedal MAC exhibited a significantly elevated risk of amputation (OR 3.64, 95% CI 1.91–6.95, *p* < .001) even after adjusting for sex, age, albumin, hemoglobin, and diabetic retinopathy status. Furthermore, in the population with DFUs, patients with both PAD and pedal MAC had twofold higher risk of amputation compared to those with only PAD but without pedal MAC (OR 2.05, 95% CI 1.10–3.82, *p =* .024). (Table 4).

**TABLE 4:** Multivariate logistic regression of risk factors of amputation in patients with diabetic foot ulcers.

	OR	95% CI	*p* value
PAD_MAC			
Normal	–	–	–
Pedal MAC alone	1.75	0.53–5.74	.354
PAD only	1.77	1.17–2.69	.007
PAD + pedal MAC	3.64	1.91–6.95	<.001
*p* value for trend	1.42	1.19–1.70	<.001
Sex	1.47	0.99–2.18	.051
Age	1.00	0.98–1.02	.853
Albumin	0.98	0.96–1.01	.317
Hemoglobin	0.99	0.98–1.00	.107
Diabetic retinopathy			
Normal	–	–	–
Mild–moderate	1.47	0.99–2.16	.052
Severe	2.10	1.13–3.89	.019
*p* value for trend	1.47	1.13–1.92	.005

*Note*: Severe diabetic retinopathy, macular edema, severe nonproliferative diabetic retinopathy, and proliferative diabetic retinopathy.

Abbreviations: CI, confidence interval; MAC, medial arterial calcification; OR, odds ratio; PAD, peripheral artery disease; PAD_MAC, a variable generated as a representative of the presence of PAD and pedal MAC.

### 3.2 Mortality

**FIGURE 1:**
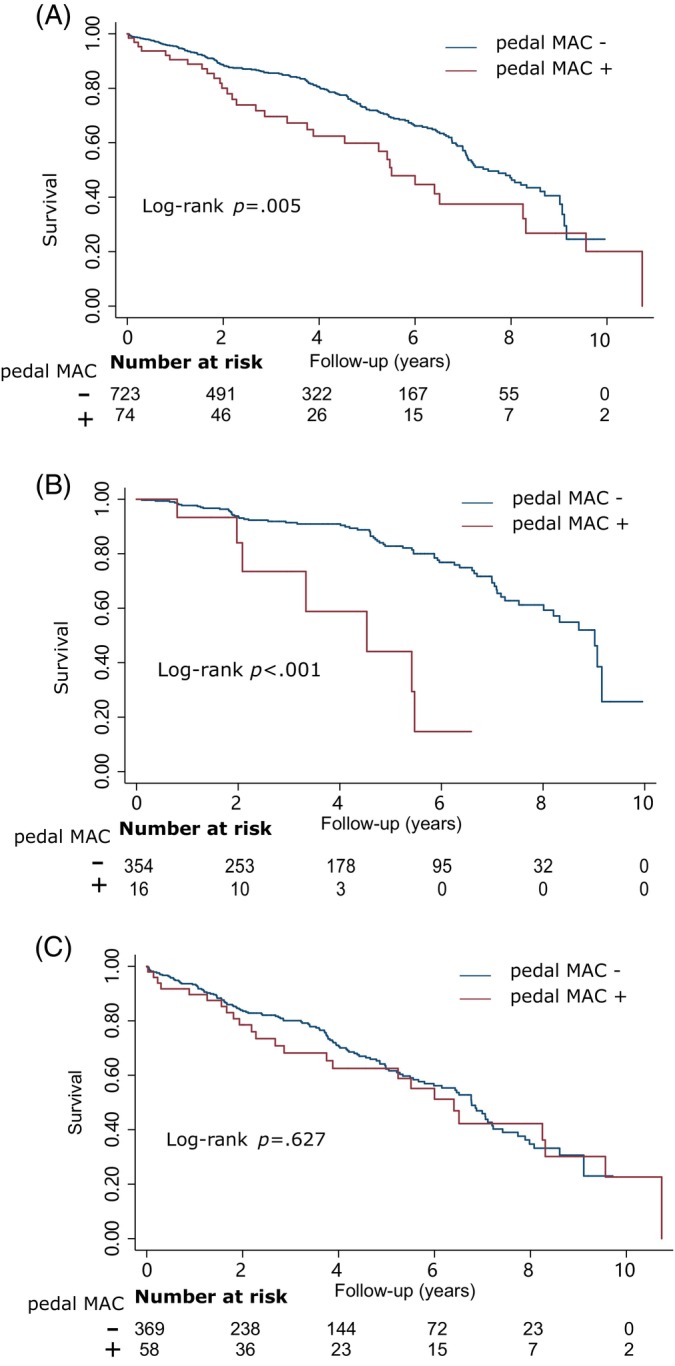
Kaplan–Meier plots of mortality by pedal medial arterial calcification. Kaplan–Meier plots of mortality of all patients with DFUs (A), patients with DFUs but without PAD (B), patients with both DFUs and PAD (C). MAC, medial arterial calcification; PAD, peripheral artery disease.

During a median follow‐up period of 46 months (IQR 23–72 months), a total of 246 (30.87%) patients experienced mortality, and an additional 182 patients were lost to follow‐up. In the Kaplan–Meier survival analysis, pedal MAC demonstrated a significant association with all‐cause mortality (log‐rank *p* = .005, Figure 1A). However, upon conducting a comprehensive data analysis, it was observed that the association between pedal MAC and mortality was solely evident in patients presenting with DFUs but without PAD (log‐rank *p* < .001, Figure 1B). The Cox model consistently demonstrated a significant association between pedal MAC and mortality in both the crude model (hazard ratio [HR] 5.60, 95% CI 2.61–12.02, *p* < .001) and after adjusting for sex, age, coronary artery disease, hemoglobin, and albumin (HR 4.26, 95% CI 1.90–9.52, *p* < .001) among patients with DFUs but without PAD. The association between patients with DFUs and with PAD was not statistically significant (log‐rank *p* = 0.627, Cox model HR 1.09, 95% CI 0.67–1.79, *p* = .718, Figure 1C).

## 4 DISCUSSION

In this study, we observed a significant association between pedal MAC and amputation in patients with DFUs. Furthermore, even among patients with DFUs and PAD, the inclusion of pedal MAC provided additional predictive value for amputation risk. Additionally, our findings revealed that pedal MAC was linked to an elevated mortality risk in patients with DFUs but without PAD.

The presence of pedal MAC was found to be associated with amputation in patients with DFUs. Specifically, a study conducted by Liu et al demonstrated that the severity of pedal arterial calcification score was significantly correlated with the occurrence of major amputation in patients suffering from chronic limb‐threatening ischemia.^14^ The severity of MAC was found to be associated with major limb adverse events in subjects undergoing angioplasty with a low wound grade, as demonstrated by Skolnik and colleagues study.^18^ Furthermore, in the population with CKD, severe MAC was shown to exacerbate the limb outcomes of DFUs.^16^ The findings of our study have extended the existing evidence linking pedal MAC and amputation in patients with severe limb ischemia and CKD to include patients with DFUs. Furthermore, we have reinforced the notion that pedal MAC can exacerbate the negative outcomes for individuals with PAD. Thus, even among patients with PAD and DFUs, pedal MAC demonstrates predictive value for assessing limb outcome.

Prior to the introduction of the novel scoring system proposed by Ferraresi et al,^17^ there was a lack of standardization in assessing pedal calcification. The observed correlation between MAC and amputation across various studies, including our own investigation, suggests its potential as a dependable diagnostic tool. Despite being widely regarded as effective for predicting PAD, ABI may exhibit reduced accuracy under specific circumstances, particularly when patients present with concurrent MAC involvement; however, it should be noted that MAC frequently coexists with PAD. Therefore, alternative approaches should be employed to complement the limitations of ABI. A study conducted by Skolnik et al demonstrated that lower extremity calcification scoring serves as a superior tool for predicting amputation risk.^18^ Furthermore, the assessment of MAC through plain radiographs is both straightforward and reproducible. Consequently, MAC could provide additional predictive value for limb outcomes beyond ABI.

Although the pathogenesis of tissue necrosis induced by MAC remains unclear, it is speculated that calcification of the tunica media results in arterial stiffening,^19^ intimal hyperplasia,^20^ progressive blood flow impairment, and subsequent thrombotic occlusion.^21^, ^22^ Pathological analyses from multiple studies have consistently identified intimal thickening, medial calcification, and thrombus formation as primary arterial lesions in PAD, particularly affecting distal arteries below the knee.^9^, ^10^ Indeed, these pathological manifestations were consistent with calciphylaxis, a condition characterized by intensely painfully ischemic necrosis of the skin.^21^ The presence of similar pathological findings in both limb gangrene caused by PAD and calciphylaxis suggests that disorders involving MAC in vessels of varying sized might share similar pathogenic mechanisms.

In terms of PAD treatment, revascularization is a crucial option for restoring tissue perfusion in patients with DFUs and PAD.^23^ However, this approach is not suitable for patients with MAC.^24^ The presence of thromboembolic phenomenon suggests that antithrombotic agents might play an important role in reducing limb events associated with PAD.^25^ In the COMPASS (Cardiovascular Outcomes for People Using Anticoagulation Strategies) trial, combination therapy with rivaroxaban and aspirin demonstrated a significant 46% reduction (*p* = .005) in major limb events compared to aspirin monotherapy in patients with PAD.^26^ Similarly, the VOYAGER PAD trial revealed that rivaroxaban plus aspirin significantly decreased the composite outcome of acute limb ischemia, major amputation for vascular causes, myocardial infarction, ischemic stroke, or death from cardiovascular causes when compared to aspirin alone in PAD patients following revascularization.^27^ However, the treatment of thrombus is targeted toward the later phase of the necrosis pathogenesis. Moreover, anticoagulation therapy may elevate the risk of bleeding.

Considering the pivotal role of MAC in limb ischemia, it is crucial to focus on preventing and reversing MAC to effectively reduce limb events in high‐risk patients. However, vascular calcification has not been considered a direct therapeutic target in clinical practice due to the lack of interventions capable of preventing, reversing or decelerating its progression.^28^ Currently, numerous interventions are being investigated in clinical trials, encompassing vitamin D and vitamin K supplementation, calcimimetics administration, phosphate binder usage, magnesium supplementation, sodium thiosulfate treatment, SNF472 application, bisphosphonates therapy initiation, denosumab administration as well as use of tissue‐nonspecific alkaline phosphatase inhibitors and alterations in dialysis techniques.^29^

In this study, we also observed that pedal MAC was associated with an increased risk of mortality in patients with DFUs, specifically in those with DFUs but without PAD. Traditionally, PAD is recognized as a manifestation of atherosclerosis, with approximately 60% of PAD patients having ischemic heart disease, and 30% having cerebrovascular disease.^30^ PAD is commonly considered an indicator of obstructive atherosclerotic disease,^31^ suggesting that atherosclerosis might be the primary cause of mortality in patients with PAD and that pedal MAC might not provide additional value. However, for patients without PAD, pedal MAC is associated with an increased risk of death, likely due to the high coincidence of ESRD and the elevated limb adverse events risk.

Our study has several limitations. First, this was a single‐center study conducted at a tertiary diabetic foot care center. Therefore, it is crucial to validate the findings in multiple centers worldwide for enhanced generalizability and robustness of the results. Second, the sample size of patients with only pedal MAC but without PAD was relatively small. Thus, further investigations involving larger populations are warranted to ascertain whether pedal MAC alone could potentially exacerbate limb outcomes in individuals with DFUs. Third, the study population was limited to individuals with DFUs. Further investigations are warranted to determine the potential impact of MAC in small and midsized vessels on limb outcomes in the general population. Fourth, logistic regression was employed to assess the association between pedal MAC and amputation; however, it is important to consider competing risk of death when analyzing amputation outcomes. Failure to adjust for competing risk of death and reliance solely on logistic regression may introduce bias.

The findings have significant implications for clinical practice. The pedal MAC can be easily evaluated using foot radiographs and exhibits robust predictive value for major amputation in patients with DFUs, thus serving as a potent adjunct for outcome stratification. However, currently there is no existing grading system for diabetic foot that incorporates this aspect. If widely validated, pedal MAC could be adapted for the prediction of amputation in DFUs. Furthermore, the findings of this study underscore the imperative to develop interventions aimed at preventing, decelerating, and potentially reversing MAC progression to mitigate amputation risk. Nevertheless, our comprehension of MAC pathogenesis remains limited due to the dearth of robust in vivo models. Most studies investigating vascular calcification employ models of atherosclerotic disease, whereas the role of MAC has now been established in the development of critical limb ischemia.^32^ Therefore, future research endeavors should focus on elucidating the pathogenesis underlying MAC initiation, progression and subsequent tissue ischemia and necrosis.

## 5 CONCLUSIONS

Pedal MAC was found to be significantly associated with amputation in patients with DFUs. Even in patients with DFUs and PAD, pedal MAC demonstrated additional predictive value for amputation. Furthermore, pedal MAC was linked to an increased risk of mortality in patients with DFUs but without PAD. Therefore, further studies are warranted to develop interventions aimed at slowing or reversing the progression of pedal MAC and ultimately reducing the incidence of amputations.

## AUTHOR CONTRIBUTIONS

Lihong Cheng: study design, data collection, statistical analysis, interpretation of the results, and manuscript writing. Dawei Chen, Hongping Gong, Chun Wang, Yun Gao, Yan Li, Weiwei Tang, Panpan Zha: data collection, and analysis. Xingwu Ran: study design and interpretation, critical revision of the manuscript. All authors have read and approved the final version of the manuscript.

## FUNDING INFORMATION

This research was supported by the West China Nursing Discipline Development Special Fund Project, Sichuan University (Grant No.: HXHL20005), the 1·3·5 Project for Disciplines of Excellence, West China Hospital, Sichuan University (Grant No.: ZYGD18025), the Health Commission of Sichuan Province (Grant No.: 23LCYJ042) and the Science and Technology Bureau of Sichuan Province (Grant No.: 2021JDKP004).

## DISCLOSURE

The authors declare that they have no competing interests.

## CONSENT FOR PUBLICATION

The manuscript was approved by all authors for publication.

## Data Availability

The datasets used and/or analyzed during the current study are available from the corresponding author on reasonable request.
